# Associations Between Parental Alexithymia and Family Dynamics in Autism Spectrum Disorder

**DOI:** 10.3390/healthcare13040373

**Published:** 2025-02-10

**Authors:** Radoslav Kosić, Daniela Petrić, Inge Vlašić-Cicvarić, Tanja Kosec

**Affiliations:** 1 Department of Neurology and Child Psychiatry, Clinic for Pediatrics, University Hospital Center Rijeka, Krešimirova 42, 51000 Rijeka, Croatia; 2 Faculty of Health Studies, University of Rijeka, V.C. Emina 5, 51000 Rijeka, Croatia; 3 Department of Child and Adolescent Psychiatry, Clinic for Psychiatry, University Hospital Center Rijeka, Krešimirova 42, 51000 Rijeka, Croatia; daniela.petric@icloud.com (D.P.); tanjakosec2@yahoo.com (T.K.); 4 Faculty of Medicine, University of Rijeka, Braće Branchetta 20, 51000 Rijeka, Croatia; inge_v_c@yahoo.com; 5 Centre for Clinical and Health Psychology, University Hospital Center Rijeka, Krešimirova 42, 51000 Rijeka, Croatia

**Keywords:** autism spectrum disorder, alexithymia, family functioning, parental stress, social support, emotional regulation in parents, family flexibillity, ASD and family dynamics

## Abstract

Background/Objectives: Alexithymia is a condition marked by difficulties in identifying and expressing emotions, rooted in both physiological and behavioral mechanisms. The aim of this study was to investigate the relationship between parental alexithymia and family functioning in families of children with Autism Spectrum Disorder (ASD) compared to families of typically developing children (TD). Methods: The study sample included parents of children with ASD (*n* = 120) and a control group of parents of typically developing children (*n* = 120). A comprehensive set of self-report instruments was used to evaluate alexithymia levels, parental stress, family experience, resilience, cognitive emotion regulation, social support, and family flexibility and cohesion. Results: The analysis revealed that parental alexithymia in families of children with ASD was directly associated with lower levels of family flexibility and cohesion, independent of increased stress or reduced family resilience. Furthermore, the findings indicate that alexithymia in parents is directly linked to reduced family cohesion in ASD families. Conclusions: These results highlight the significant role of parental alexithymia in shaping family dynamics and underscore the necessity for targeted interventions that emphasize emotional skill-building, adaptive coping mechanisms, and resilience to stressful events. This research enhances the understanding of parental alexithymia’s effect on family functioning in the context of ASD.

## 1. Introduction

Alexithymia is a psychological construct characterized by difficulties in identifying and describing one’s own emotions, a lack of symbolic thinking, and limited emotional awareness. It is not formally recognized as a disorder in diagnostic manuals but is instead viewed as a personality trait that can vary in degree across individuals. While it is not classified as a specific disorder, research has shown that alexithymia is associated with an increased risk of psychopathology, particularly in individuals with certain mental health conditions [1,2]. 

Estimates suggest that certain characteristics of alexithymia are present in approximately 5–10% of the general population [3], while in the psychiatric patient population the prevalence of this disorder is significantly higher. High rates of alexithymia (ranging from 20% to 60%) have been reported in patients with psychosomatic disorders, mood disorders and anxiety disorders [4], while in autism spectrum disorders alexithymia occurs in about 50% of the cases [5]. Etiological factors are categorized into two main areas: neurobiological, corresponding to primary alexithymia, and psychosocial, relating to secondary alexithymia (alexithymic characteristics resulting from developmental delays, psychological trauma in childhood or later in life, sociocultural or psychodynamic factors) [6].

The presence of alexithymia, and other characteristics of autism spectrum disorder (ASD), is often detected in parents and relatives of people with clinically diagnosed autism, and this phenomenon is denoted in the literature by the term “Broad Autism Phenotype” (BAP) [7]. Previous studies suggest there is a correlation between parental alexithymia and the expression of emotions in children with ASD [8]. Parents of children with ASD often struggle to recognize, describe, and differentiate feelings; consequently, they communicate much less and have fewer social interactions with the child, compared to the parents of a typically developing (TD) child. The above said difficulties result in insufficient stimulation of the child’s development, they hinder the recognition of the child’s needs, impede the initiation of early intervention programs, and ultimately, worsen the overall functioning of the family [8,9,10,11].

Numerous studies have shown that parents of children with ASD experience higher levels of stress [8], anxiety [12], and depression [13,14]; their social contacts are significantly reduced [15] and they have poorer physical health compared to the general population and parents of children with other chronic health conditions (e.g., Down syndrome and cerebral palsy) [13,16,17]. It is known that formal and informal social support positively impacts the mental health and general well-being of caregivers of children with ASD [18]. Moreover, it can act as a protective factor in dealing with the daily challenges of caring for a child with ASD [19,20]. Another important factor is family functioning, which can generally be defined as a way in which family members interact with each other [9], respond to, and treat one another [21,22]. Olson et al. [23] identified two basic dimensions underlying family functioning: cohesion and flexibility. Cohesion refers to the emotional connection between family members, while flexibility denotes the capacity to adapt family structure based on events that occur during the life cycle. Levels of family cohesion and flexibility in families with children with ASD, according to some studies, are lower compared to families of TD children [24]. Research by Gau et al. [25] highlighted that parents of children with ASD exhibited higher levels of psychopathology and a lower dyadic consensus compared to parents of TD children. Additionally, the study found that mothers of children with ASD reported experiencing lower marriage satisfaction, as well as challenges in attachment, family flexibility, and cohesion, in comparison to mothers of TD children.

With consideration to research findings on the prevalence of BAP [7], we conducted a study to further investigate the construct of alexithymia in parents of children with ASD and parents of TD children, as a potentially very important component in general family functioning. Our hypothesis was that alexithymia in parents of children with ASD could be one of the key factors contributing to poorer family functioning. We conducted the study on both parents, unlike most previous studies that had mainly focused on the experiences of mothers, or the gender of parents had not been important for the research itself. This means that opinions, reflections, and general experience of fathers were less known. We presumed that the level of alexithymia in parents of children with ASD would be significantly higher compared to parents of TD children. We also presumed that alexithymia in parents of children with ASD was directly related to poorer family functioning (family experience with autism, and that it was also associated with lower levels of cognitive emotion regulation and lower levels of social support). Finally, we hypothesized that alexithymia in parents of children with ASD was directly related to a lower level of family flexibility and cohesion, but was also associated with higher levels of stress and lower levels of family resilience. The results suggest that alexithymia in parents has a direct negative impact on levels of family cohesion in families of children with autism spectrum disorder.

## 2. Materials and Methods

### 2.1. Participants

The study included 240 respondents, of which 60 pairs of parents of children diagnosed with ASD and 60 pairs of parents of TD children. The sample size was determined by considering the regression analyses with 8 predictors. The research was based on the statistical power of 80%, with a significance level (*p* value) limit of 0.05 and a moderate effect size of f2 = 0.15. For these parameters, the minimum sample size was 109 respondents per group, and the calculation additionally took into account 10% of potentially improperly completed questionnaires.

The inclusive criteria for parents of children with ASD were as follows: children who had received a medical diagnosis of Autism Spectrum Disorder based on the DSM-5 criteria; children living at home with both of their parents; children aged between 3 and 15 years; children who had undergone a diagnostic assessment and/or were enrolled in a special or inclusive school, or had received therapeutic treatment at the Rijeka Autism Centre; children whose parents did not have any accompanying conditions (such as serious physical illness, hearing or vision impairments, etc.); children from families without any developmental disorders or serious health issues; children whose parents were partners and resided together; children whose both parents completed the questionnaires; both parents were the biological parents of the child.

For parents of children with typical TD, the inclusion criteria were as follows: none of their children had been diagnosed with any developmental disorders, intellectual disabilities, or serious health conditions, the child was aged between 3 and 15 years; parents did not have any comorbid conditions (e.g., serious physical illnesses, hearing or vision impairment; the parents were partners living together in the same household; both parents completed the required questionnaires, both parents were the biological parents of the child.

These inclusion criteria were based on prior research involving parents of children with typical development and developmental disorders, as well as established guidelines for participant selection in family functioning studies [26,27].

Exclusion criteria, applicable to both groups (parents of children with autism spectrum disorder [ASD] and parents of children with TD), included the following: presence of severe psychological disorders in parents (e.g., psychosis); presence of severe physical illnesses in parents that could hinder participation in the study; parents who declined participation in the study.

These exclusion criteria were defined to ensure sample homogeneity and to minimize confounding factors that might compromise the validity of the findings, as recommended in relevant methodological literature [28].

The two groups of parents were not statistically different in terms of characteristics such as the age and gender of the child. The data was collected from 1 October 2022 to 30 September 2023.

### 2.2. Methodology

The inclusion of parents in the first (clinical) group was carried out using the database of the Clinical Hospital Centre Rijeka and the Rijeka Autism Centre. The target sample were the parents whose children had undergone multidisciplinary assessment at the Rijeka Clinical Hospital Centre or attended the Rijeka Autism Centre and had received a diagnosis of ASD. The second (control) group comprised parents of TD children, who were recruited in collaboration with the pediatrician of the Health Centre of Primorje-Gorski Kotar County. The consent was obtained from team managers in pediatric clinics. Upon obtaining consent from the parents, the researcher established contact with the parents by phone. Once information was provided and oral consent was obtained, an appointment time for examination was arranged. Parents were asked to sign the informed consent to participate in the research. The instructions were given to parents individually, through a standardized protocol. The instructions were provided by the researcher, who was present throughout the entire process of completing the questionnaires, clarifying any potential ambiguities. It took around an hour and a half to complete the questionnaire. The completed questionnaires were reviewed by the researcher and, in cases where missed or unclear answers were found, parents were requested to provide clarification. Each respondent was assigned a unique identification number, guaranteeing the security of the respondent’s identity. All questionnaires were sealed in envelopes that were not opened until it was time for statistical analysis.

### 2.3. Procedures and Analyses

The researchers developed a custom questionnaire specifically for this study to gather detailed demographic data. The questionnaire included items addressing gender, age, education level, occupation, employment status, parental physical and mental health conditions, total family size, the number of children in the family, the child’s age and gender, and instances of parental absence from work due to the child’s illness. In addition to the demographic questionnaire, the study employed several validated self-assessment tools to provide a comprehensive analysis. These included the Family Resilience Assessment Scale, the Autism Family Experience Questionnaire, the Questionnaire for Measuring Stressors and Intensity of Parental Stress, the Family Adaptability and Cohesion Evaluation Scale IV, the Toronto Alexithymia Scale 20, the Cognitive Emotion Regulation Questionnaire, and the Multidimensional Scale of Perceived Social Support.

The Family Resilience Assessment Scale (FRAS) was utilized to evaluate the resilience of families in the face of challenges (FRAS; [29]). This 54-item questionnaire, divided into six key factors, measured aspects such as family communication and problem-solving, resource utilization, family cohesion, maintaining a positive attitude, spirituality, and the ability to find meaning in adversity. Each item was rated on a 4-point Likert scale, with an overall reliability of α = 0.96. Subscale reliabilities were equally strong, ranging from 0.70 to 0.96, based on the original validation study [29].

To capture the unique experiences of families with autistic children, the Autism Family Experience Questionnaire (AFEQ) was used [30]. With the author’s permission, the AFEQ was translated into Croatian using a rigorous double-translation process. This 48-item questionnaire, rated on a 5-point Likert scale, assessed four domains: the experience of being a parent of an autistic child, family life, the child’s development and social relationships, and the child’s symptoms (feelings and behavior). Lower scores indicated better outcomes, with strong reliability across domains: parent (α = 0.85), family (α = 0.83), child development (α = 0.81), child symptoms (α = 0.79), and total score (α = 0.92), as reported in the original validation study [30].

Parental stress was evaluated using the Questionnaire for Measuring Stressors and Intensity of Parental Stress [31]. This comprehensive tool included 65 items rated on a 3-point Likert scale. These items were grouped into 13 subscales under three main categories: child characteristics (e.g., difficulty, lack of adaptation, health), parent-child interactions (e.g., unfulfilled expectations, attachment, discipline, communication), and parent/social network characteristics (e.g., incompetence, lack of support, financial challenges, spousal relationship). The overall reliability of the questionnaire was reported as α = 0.96 in the validation study conducted by Profaca and Arambašić [31].

Family adaptability and cohesion were assessed using the Family Adaptability and Cohesion Evaluation Scale IV (FACES IV) [32]. This 62-item scale, grounded in the Olson Circumplex Model, evaluated family cohesion and flexibility. Participants rated items on a 5-point Likert scale, with higher scores reflecting better family functioning. The scale demonstrated strong reliability, ranging from 0.70 to 0.90, as documented in the original validation study [32].

To explore emotional awareness, the Toronto Alexithymia Scale 20 (TAS-20) was employed [33]. This 20-item instrument assessed three dimensions of alexithymia: difficulty recognizing emotions, difficulty describing emotions, and externally-oriented thinking. Scores ranged from 20 to 100, with higher scores indicating greater levels of alexithymia. Categories included no alexithymia (<52), borderline alexithymia (52–60), and alexithymia (≥61). The scale showed high internal reliability (α = 0.86 overall), with subscale reliabilities ranging from α = 0.71 to 0.79, based on the original validation study [33].

The Cognitive Emotion Regulation Questionnaire (CERQ) [34] is a multidimensional tool designed to assess cognitive strategies individuals use to manage negative events or situations. To ensure cultural relevance, Soldo and Vulić-Prtorić [35] adapted the questionnaire for the Croatian context. It comprises 36 items grouped into nine subscales, each containing four statements rated on a 5-point Likert scale. The CERQ is suitable for individuals aged 12 and older, as it assumes participants at this age possess the cognitive capacity to comprehend the items. In this study, participants reported the frequency with which they used each cognitive strategy following an unpleasant experience. Scores for each strategy were calculated by summing the responses to the corresponding items, resulting in subscale scores ranging from 4 to 20. Higher scores indicate more frequent use of a particular strategy, while lower scores suggest infrequent use. The CERQ is versatile, allowing researchers to examine either general coping styles or responses to specific events, depending on the study’s focus. The questionnaire demonstrates solid internal consistency, with Cronbach’s alpha values ranging from 0.73 to 0.89, as reported in the validation study by Soldo and Vulić-Prtorić [35].

The Multidimensional Scale of Perceived Social Support (MSPSS) [36] was adapted for the Croatian cultural context by Medved and Keresteš [37]. This scale comprises 12 statements designed to evaluate an individual’s perception of support from three key sources: family, friends, and a significant other. Each source is represented by four statements, such as “My family is willing to help me make decisions” (family), “I have friends with whom I can share my joys and sorrows” (friends), and “I have a special person who is a real source of comfort to me” (significant other). Participants rated their agreement with each statement on a 7-point Likert scale, with higher scores reflecting greater perceived social support from the respective source. Subscale scores were calculated by summing the responses for the relevant items. The MSPSS demonstrated excellent reliability in its validation study, with an overall Cronbach’s alpha of α = 0.93. The reliability of the three subscales was also high, ranging from α = 0.89 to 0.91 [37].

### 2.4. Statistical Data Analysis

First, the normality and symmetry of the distributions of all continuous variables were tested using the Kolmogorov-Smirnov test, followed by the calculation of skewness and kurtosis coefficients. The results revealed that the distributions of most variables significantly deviated from normality; however, the calculated skewness and kurtosis coefficients did not indicate substantial asymmetry (Table 1). It is generally accepted that parametric statistical procedures can be used when skewness and kurtosis values fall within the range of ±2 [38]. Some authors further suggest that a distribution is not significantly different from normal if skewness values lie between −2 and +2 and kurtosis values are between −7 and +7 [39]. In this study, since the skewness and kurtosis coefficients were within the range of −2 to +2, parametric statistical procedures were applied in the majority of subsequent analyses.

Pearson’s correlation coefficient and independent samples *t*-tests were used to compare two groups of participants, while analysis of variance (ANOVA) was conducted to examine interaction effects between two or more independent variables. Additionally, the Chi-square test was employed to compare categorical variables (e.g., employment status, presence of physical or mental illness), and the Mann-Whitney U-test was used to assess differences in the number of family members and days of sick leave between the two groups.

Multivariate regression analysis was performed to examine the relationship between parents’ alexithymia and measures of family functioning. Predictor variables included the score on the alexithymia scale, parental stress, cognitive emotion regulation, family resilience, and perceived social support. Family functioning was assessed using two scales: one measuring the experiences of families with autism and another evaluating family flexibility and cohesion.

All statistical analyses were performed using Statistica version 12.5 (StatSoft Inc., Tulsa, OK, USA). In all analyses, *p* < 0.05 was considered statistically significant.

## 3. Results

We have not established any statistically significant difference between parents of children with ASD and parents of typically developing (TD) children in terms of age (t = 1.65; *p* = 0.101) and level of education (χ^2^ = 4.38; *p* = 0.112). The average age in the group of parents of children with ASD was (M = 34.5), while in the group of parents of typically developing (TD) children, it was (M = 35.6). On average, mothers were significantly younger than fathers, both in the group of parents of children with ASD (χ^2^ = 4.65; *p* < 0.001) and in the group of parents of TD children (χ^2^ = 3.32; *p* < 0.001). The majority of parents of children with ASD (63%) and parents of TD children (53%) had completed high school.

A statistically significant difference was found in permanent employment status between parents of typically developing (TD) children, with 82% being employed, and parents of children with ASD, who were more often unemployed (14%) or only temporarily employed (30%) (χ^2^ = 20.21; *p* = 0.001).

One quarter of parents of children with ASD had been diagnosed with a physical illness, while in the group of parents of TD children the incidence of physical diseases was significantly lower (χ^2^ = 14.52; *p* = 0.001). The most frequently reported diseases among parents of children with ASD were migraine (7%), hypertension (5%) and diabetes (4%). In parents of TD children, hypertension was most common (3%), while other diseases were reported in a much smaller percentage. In addition to physical illnesses, parents of children with ASD reported a significantly higher percentage of mental illnesses (χ^2^ = 3.90; *p* = 0.048), most often anxiety (6%) and depression (5%). Parents of children with ASD were also taking permanent or occasional drug therapy to a significantly higher extent than parents of TD children (*n* = 44) (χ^2^ = 22.64; *p* = 0.001).

Parents of children with ASD had spent statistically significantly more days on sick leave in the previous 6 months due to the child’s sickness compared to parents of TD children (Mann-Whitney U = 7200.0; *p* < 0.001). The majority of parents of TD children, 80 of them (66%), had not taken any sick leave, while the remaining parents had taken sick leave due to their child’s illness for no more than two weeks. In the previous 6 months, all parents of children with ASD had been on sick leave due to the child’s illness, lasting from 2 weeks to more than a month.

Table 2 shows some of the sociodemographic characteristics between parents of children with ASD and parents of children with TD.

Parents of children with ASD had a significantly higher score in all three segments of alexithymia and in the overall Toronto Alexithymia Scale (Table 3).

Parents of typically developing children score significantly higher on balanced family flexibility and cohesion scales, while parents of children with ASD score higher on the extreme poles of unbalanced flexibility and cohesion. Additionally, parents of children with ASD report lower quality of family communication and overall satisfaction with family life (Table 4).

Parents of children with ASD rate their overall family resilience significantly lower than parents of TD children. They also report lower levels of family communication and problem-solving, cohesion, and the ability to find meaning in adversity (Table 5).

Parental stress levels are more than twice as high in parents of children with ASD compared to parents of TD children. The largest differences are observed in the domains of the child’s lack of adaptation, unfulfilled expectations, and health-related concerns, where parents of children with ASD report significantly greater stress (Table 6).

Parents of children with ASD differ significantly from parents of TD children in most cognitive emotion regulation strategies. They are more likely to use self-blame, catastrophizing, and blaming others, while parents of TD children more frequently use acceptance, positive refocusing, refocus on planning, positive reappraisal, and putting into perspective. Both groups show no significant difference in the use of rumination (Table 7).

Parents of children with ASD perceive significantly lower overall social support compared to parents of TD children, including lower levels of family support, support from friends, and support from a significant other (Table 8).

The family experience with autism scale, administered only to parents of children with ASD, revealed an average overall score of 162.52 (SD = 10.42) out of 240. Subscale scores showed moderate challenges across domains, including parenting experience, family life, child’s development and social relationships, and child’s symptoms (Table 9). 

An overview of the relationship between alexithymia and family flexibility and cohesion, taking into account the potential mediation effects of social support, cognitive emotional regulation, resilience and parental stress. Comparison of predictor models for families of children with ASD and family of TD children.

### 3.1. Family Cohesion

In families of children with ASD, a lower assessment of family cohesion is associated with female sex (r = −0.22; *p* < 0.05) and greater age of parents (r = −0.19; *p* < 0.05), whereas higher level of family cohesion is related to employment status of the parents (r = 0.20; *p* < 0.05) and greater number of family members (r = 0.25; *p* < 0.01). A higher level of alexithymia is relatively strongly associated with a lower assessment of family cohesion (r = −0.55; *p* < 0.01). The level of social support is also negatively correlated with the level of family cohesion (r = −0.19; *p* < 0.05), while the level of parental stress did not appear to be in statistically significant correlation with perceived family cohesion.

Regarding the scales of cognitive emotion regulation, only self-blame is in a statistically significant negative correlation with the level of family cohesion (r = −0.21; *p* < 0.01). Concerning family resilience, in families with ASD children, family cohesion is in a statistically significant correlation only with the ability to find meaning in adversity (r = 0.17; *p* < 0.05) (Table 10).

In families of TD children, potential predictors of family cohesion are somewhat different. Male sex (r = −0.49; *p* < 0.01) and parents’ employment status (r = 0.45; *p* < 0.01) are in a statistically significant correlation with a higher assessment of family cohesion. Unlike in families of children with ASD, in parents of TD children alexithymia is not significantly associated with the levels of family cohesion (r = 0.09; *p* > 0.05), parental stress (r = 0.03; *p* > 0.05), and social support (r = 0.01; *p* > 0.05). Furthermore, none of the family resilience scales demonstrate a statistically significant association with family cohesion. Regarding cognitive emotion regulation, self-blame (r = −0.37; *p* < 0.01), rumination (r = −0.46; *p* < 0.01) and catastrophizing (r = −0.18; *p* < 0.05) are in a statistically significant correlation with family cohesion, while refocus on planning (r = 0.20; *p* < 0.05) is positively associated with family cohesion (Table 11).

Table 12 presents the results of hierarchical regression analysis for families of children with ASD and families with TD children. The regression models include the measures and characteristics that were found to be in a statistically significant correlation with the score on the family cohesion scale.

In the context of hierarchical regression analysis, the term “not a predictor” refers to variables that did not show a statistically significant correlation with the criterion variable in the previous steps of the modeling process. Specifically, in our analysis, the regression models included only those variables that demonstrated a statistically significant correlation with the results on the family cohesion and flexibility scale in the previous step. These variables were selected based on their correlations with the criterion variable, meaning they were deemed relevant for further modeling.

This approach allows for the systematic evaluation of the contribution of each new variable in predicting the criterion outcome, while also ensuring that variables not significantly related to the outcome are not included. Decisions regarding the inclusion or exclusion of variables were based on the statistical analyses conducted in the previous steps and are clearly stated in the text, with references to the tables containing the regression analyses (Table 13, Table 14 and Table 15).

We coded the nominal variables used in the regression analyses (Table 13 and Table 15) as follows: Sex: 0 = female, 1 = male; Education level: 0 = no schooling, 1 = elementary school, 2 = high school, 3 = college, 4 = university; Number of family members: 1 = 3 members, 2 = 4 members, 3 = 5 members, 4 = 6 or more members; Employment status: 1 = employed, 2 = occasionally employed, 3 = unemployed.

This predictor model, in the group of families of children with ASD, accounts for 48.6% of the variance in family cohesion. Of the sociodemographic characteristics, in the final predictor model, variables of gender and age of parents significantly contribute to explaining the variance of criteria, while employment status and number of family members are no longer significant predictors. Women, as well as older parents, have lower scores on the family cohesion scale. Parents with higher levels of alexithymia also score lower on the family cohesion scale. Of the segments of cognitive emotion regulation, only self-blame offers a statistically significant contribution in predicting the level of family cohesion indicating that parents who are more inclined to this strategy assess family cohesion as lower. The ability to find meaning in adversity, as one of the elements of family resilience, do not prove significant in this predictor model (Table 10). The very small change in the independent contribution of alexithymia to the explanation of the variance in family cohesion in the final regression model (β (step 2) = −0.52; *p* < 0.01 vs. β (step 3) = −0.50; *p* < 0.01) does not point to any significant mediation effect of other variables. The results suggest that parental alexithymia has a direct negative effect on the level of family cohesion in families of children with ASD.

In parents with TD children, the presented predictor model explains 44.6% of the variance in family cohesion. Of the sociodemographic characteristics, in the final predictor model, gender and employment status of parents significantly contribute to explaining the variance of criteria. Women as well as unemployed parents score lower on the family cohesion assessment scale. As already mentioned, alexithymia does not make a statistically significant contribution in this model. However, cognitive emotion regulation strategies, particularly the “negative” ones, play a significant role in predicting family functioning. Parents prone to self-blame, rumination and catastrophizing assess family cohesion as lower, while those who tend to refocus on planning have better outcomes in terms of family functioning as measured by the family cohesion scale (Table 12).

### 3.2. Family Flexibility

In families of children with ASD, a lower assessment of family flexibility is associated with male sex (r = 0.46; *p* < 0.01) and a lower age of parents (r = 0.22; *p* < 0.05), while other socio-demographic characteristics are not significantly correlated with this measure of family functioning. The level of alexithymia in parents of children with ASD is also not significantly associated to the reported family flexibility. Higher levels of parental stress (r = −0.31; *p* < 0.01) and lower level of social support (r = 0.49; *p* < 0.01) are associated with a lower score in family flexibility scale, as well as a higher score in self-blame (r = −0.30; *p* < 0.01) and catastrophizing (r = −0.42; *p* < 0.01). Other cognitive emotion regulation scales, as well as family resilience scales, are not in a statistically significant correlation with the measure of family flexibility in families of children with ASD (Table 13).

In families of TD children, predictors of family flexibility are somewhat different. Male sex (r = −0.23; *p* < 0.05), younger age (r = −0.22; *p* < 0.01) and higher education status of parents (r = 0.22; *p* < 0.05) are in a statistically significant correlation with a higher assessment of family flexibility, while other socio-demographic characteristics are not significantly associated with this measure of family functioning. Furthermore, no statistically significant correlation has been established between alexithymia levels in parents of TD children and the assessments of family flexibility (r = 0.08; *p* > 0.05) and parental stress (r = 0.03; *p* > 0.05). The level of social support is found to be positively correlated with family flexibility (r = 0.26; *p* < 0.01). Regarding the scales of cognitive emotion regulation, family flexibility is found to be negatively correlated with rumination (r = −0.28; *p* < 0.01), catastrophizing (r = −0.18; *p* < 0.05) and blaming others (r = −0.19; *p* < 0.05). Family communication scale and problem solving (r = 0.18; *p* < 0.05), as well as family cohesion (r = 0.29; *p* < 0.01), are in a positive correlation with the assessment of family flexibility (Table 14).

Table 15 presents the results of hierarchical regression analysis for families of children with ASD and families with TD children. The regression models include the measures and characteristics that were found to be in a statistically significant correlation with the score on the family flexibility scale.

In families of children with ASD, this predictor model accounts for 54% of the variance observed in family flexibility. Of the sociodemographic characteristics, in the final predictor model, only the variable of the parents’ sex makes significant contribution in explaining the variance of criteria, while the variable of age does not have a significant predicting role. Lower levels of parental stress and self-blame, as well as higher levels of social support, are significant predictors of a better score on the family flexibility scale in families of children with ASD (Table 15).

In parents of TD children, the presented predictor model accounts for 60.7% of the variance in family flexibility. Regarding the sociodemographic characteristics, in the final predictor model, only the education level of parents has a significant impact in explaining the variance of criteria. Social support does not appear to have a statistically significant impact, however, a higher level of parental stress, as well as a higher level of catastrophizing and blaming others are significant predictors of a worse outcome on the scale of family flexibility. On the other hand, parents who score higher on the scale of putting in perspective and a higher score on the scale of family cohesion achieve a higher score in family flexibility scale (Table 15).

Alexithymia does not emerge as a significant predictor of family flexibility in this predictor model, either in the families of children with ASD or in the families of TD children.

## 4. Discussion

This observational study examined the correlation of alexithymia levels in parents of children with ASD and parents of TD children with measures of family functioning (flexibility and cohesion), taking into account the potential mediation effects of parental stress, cognitive emotion regulation, family experience with autism, family resilience, and social support.

This study highlights significant differences in the levels of alexithymia between parents of children with ASD and those with TD children, emphasizing its negative relationship with family functioning. Elevated alexithymia among parents of children with ASD is linked to reduced family cohesion and flexibility, underscoring the necessity of addressing emotional literacy within these families. Consistent with previous research [40,41,42], this finding supports the need to integrate targeted programs that enhance emotional awareness and skills among parents.

While it is true that individuals with high levels of alexithymia may face difficulties in identifying and describing emotions, this does not imply that their self-reported assessments of family cohesion and flexibility are entirely inaccurate. Parents with alexithymia might provide generalized evaluations rather than nuanced insights, but these general impressions still offer valuable information about family dynamics. Moreover, although alexithymia affects the specificity of cognitive emotion regulation strategies reported by parents, the significant association between self-blame and reduced family cohesion indicates that patterns of emotion regulation can still be identified despite challenges in emotional awareness [40,43].

A noteworthy finding is the lack of a significant relationship between alexithymia and family flexibility in families of children with ASD. However, increased parental stress and reduced social support are strongly associated with diminished flexibility and cohesion, consistent with previous studies [44,45,46]. The results emphasize the necessity of fostering resilience and enhancing social support networks to mitigate these adverse effects [47,48].

Parents of children with ASD report higher stress levels and perceive lower support from family, friends, and significant others compared to parents of TD children. These disparities highlight the importance of systemic interventions aimed at strengthening social support and improving overall family well-being. Research suggests that such interventions can positively effect family cohesion and adaptability, leading to better outcomes for families [44,49].

Sociodemographic factors also play a significant role. Parental age, gender, employment status, and family size correlate with varying levels of family cohesion and flexibility. Specifically, older parental age and female gender are associated with lower cohesion, while employment and larger family size are linked to higher cohesion [50]. These findings suggest the need for tailored interventions that address the unique challenges faced by different parental subgroups.

The findings align with prior research, which suggests that self-blame and catastrophizing are negatively associated with family cohesion, while adaptive strategies, such as planning and positive reframing, promote better family functioning [51,52]. It is essential to promote these adaptive strategies through counseling and targeted interventions.

In conclusion, this study underscores the complex interplay of parental alexithymia, stress, and social support in shaping family dynamics in families of children with ASD. Addressing parental emotional literacy, enhancing social support networks, and promoting adaptive coping mechanisms are critical steps toward improving family cohesion, flexibility, and overall quality of life. Holistic approaches that integrate emotional, social, and systemic support for parents and families of children with ASD are paramount [53,54,55].

### 4.1. Limitations

Although the results of the study provide valuable insights, there are certain limitations that should be considered. First, the study relied on self-report measures, which may be subject to social desirability bias and may not fully reflect actual family functioning, as participants may report behaviors they perceive as socially acceptable. Additionally, self-reports may not capture the complexity of family dynamics, and future research could benefit from using multiple sources of information. Second, the study employed a cross-sectional design, which does not allow for the establishment of causal relationships or the monitoring of changes in family functioning over time. This design thus cannot confirm whether parental alexithymia directly causes changes in family dynamics. Third, the use of the TAS-20 scale to assess alexithymia, while commonly used, may not be the most precise instrument in all cases, and employing the Toronto Structured Interview for Alexithymia (TSIA) could provide a deeper understanding of the psychological aspects of alexithymia [56]. Fourth, the study was conducted in a specific geographic and cultural context, which limits the generalizability of the results to broader populations. For wider applicability, future research should include a diverse population from different cultural and societal environments. Finally, the study focused on parents of children with PSA and TR, and did not include parents of children with other neurodevelopmental disorders or psychological difficulties, so future studies should expand the sample to include parents of children with different diagnoses. Additionally, while the analysis of data normality using measures such as skewness and kurtosis provided useful insights, it is recommended to also perform the Shapiro-Wilk test for normality to ensure a more accurate assessment of the data distribution.

### 4.2. Practical Implications and Recommendations for Future Research

The findings of this study highlight the importance of addressing parental emotional well-being and family dynamics in families of children with ASD. Developing interventions focused on reducing alexithymia and stress, while enhancing communication and social support, could significantly improve family functioning [42]. Emotion-focused therapy programs aimed at increasing parental emotional awareness and expression may help reduce the negative impact of alexithymia on family cohesion and adaptability [49]. Mindfulness-based stress reduction techniques and family counseling could also mitigate heightened stress levels experienced by parents, strengthening family relationships [51].

In addition, establishing support networks and providing skill-building workshops in adaptive coping strategies would empower families and foster resilience [52]. These interventions collectively contribute to promoting healthier family environments and improving the overall quality of life for families of children with ASD.

To build on these practical interventions, future research should focus on longitudinal studies to examine how parental alexithymia impacts family functioning and child development over time [45]. Investigating the efficacy of interventions targeting alexithymia, stress, and resilience will be crucial for improving family outcomes. Furthermore, studies should explore the role of cultural, societal, and demographic factors in influencing the relationship between parental emotional challenges and family dynamics. This would broaden the understanding of these issues across diverse populations.

Including more heterogeneous samples, considering socioeconomic, ethnic, and geographical diversity, would enhance the generalizability of findings. Additionally, exploring the interplay between parental emotional difficulties and child developmental outcomes could provide valuable insights into how family dynamics affect children’s well-being. By addressing these areas, future research can contribute to the development of more tailored, evidence-based interventions for families of children with ASD, promoting healthier and more resilient family systems.

## 5. Conclusions

This study highlights the significant role of parental alexithymia, stress, and social support in shaping family dynamics within families of children with ASD. The results show that parents of children with ASD face heightened emotional and social challenges, with higher levels of alexithymia being strongly associated with reduced family cohesion and adaptability. These findings emphasize the necessity of addressing difficulties in emotional regulation and stress within these families, as they significantly affect family functioning and well-being.

Given the identified relationship between alexithymia and family cohesion, targeted interventions focusing on emotional literacy, stress management techniques, and resilience-building are crucial to mitigating the negative effects of these psychological challenges. Emotion-focused programs, mindfulness techniques, and family counseling may improve emotional regulation and strengthen family relationships. Additionally, supporting parents in developing adaptive coping strategies would further contribute to enhancing family resilience and overall functioning.

The study also underscores the need for continued research on the long-term effects of parental alexithymia and stress on family dynamics, including the effect on child development. Future research should focus on examining the efficacy of interventions designed to enhance emotional awareness and adaptive coping mechanisms. It is essential to explore how cultural, societal, and individual factors shape the psychological challenges faced by these families. By investigating these variables, we can develop more tailored interventions that address the specific needs of parents and children within diverse family systems.

## Figures and Tables

**Table 1:** Presentation of characteristics of the distributions of all continuous measures included in the analysis.

*n* = 240	K-S Test	*p*	Skewness Coefficient	Kurtosis Coefficient
Age	0.07	0.001	0.28	−0.60
Balanced Family Cohesion	0.12	0.001	−0.02	−1.27
Balanced Family Adaptability	0.17	0.001	0.01	−1.42
Unbalanced Cohesion—Disengaged	0.16	0.001	0.09	−1.54
Unbalanced Cohesion—Enmeshed	0.14	0.001	0.07	−1.09
Unbalanced Adaptability—Rigid	0.16	0.001	0.22	−1.06
Unbalanced Adaptability—Chaotic	0.11	0.001	0.74	0.71
Family Communication	0.22	0.001	−0.68	−0.70
Family Satisfaction	0.15	0.001	−0.72	−0.54
Family Communication and Problem-Solving	0.12	0.001	−0.25	−1.17
Family Connectedness	0.11	0.001	−0.43	−0.48
Ability to Find Meaning in Adversities	0.11	0.001	−0.28	−0.84
Child’s Demands	0.18	0.001	−0.30	−1.28
Child’s Maladaptation	0.15	0.001	0.18	−1.37
Child’s Health	0.19	0.001	0.18	−1.53
Unmet Expectations	0.16	0.001	0.00	−1.35
Attachment to Child	0.15	0.001	0.17	−0.99
Child Discipline	0.11	0.001	−0.02	−1.02
Communication with Child	0.15	0.001	−0.09	−0.90
Incompetence	0.11	0.001	−0.15	−0.34
Lack of Support	0.12	0.001	−0.19	−1.16
Gender Role Constraints	0.16	0.001	−0.26	−0.88
Spousal Relationships	0.12	0.001	0.07	−1.01
Material Situation	0.11	0.001	−0.08	0.15
Demands of Other Roles	0.13	0.001	−0.35	−0.50
PARENTAL STRESS Total	0.15	0.001	−0.00	−1.36
Self-Blame	0.16	0.001	−0.42	−0.90
Acceptance	0.16	0.001	−0.34	−0.04
Rumination	0.14	0.001	0.16	−0.89
Positive Refocusing	0.14	0.001	−0.19	−1.23
Planning Redirection	0.17	0.001	−0.02	−1.48
Positive Reappraisal	0.14	0.001	−0.05	−1.21
Putting into Perspective	0.16	0.001	−0.48	−0.45
Catastrophizing	0.18	0.001	−0.47	−0.96
Blaming Others	0.14	0.001	−0.16	−0.15
Support from Family	0.14	0.001	−0.28	−0.87
Support from Friends	0.11	0.001	−0.19	−0.98
Support from Significant Others	0.15	0.001	−0.19	−1.06
MSPSS Total	0.10	0.001	−0.20	−1.12
Experience of Being a Parent of a Child with Autism	0.17	0.001	−0.64	0.19
Family Life	0.14	0.001	−0.14	−0.32
Child’s Development, Understanding, and Social Relations	0.18	0.001	−0.47	−0.94
Child’s Symptoms (Feelings and Behaviors)	0.19	0.001	−0.23	−1.31
AFEQ Total	0.14	0.001	−0.49	−0.63
Difficulty Describing Feelings	0.13	0.001	0.09	−0.70
Difficulty Identifying Feelings	0.12	0.001	0.33	−1.08
Externalized Thinking	0.13	0.001	0.30	0.11
TAS Total	0.13	0.001	0.35	−0.86

K-S test: Kolmogorov-Smirnov test; *p*: *p* values.

**Table 2:** Demographic Characteristics of Participants.

Characteristic	ASD *n* (%) *n* = 120	TD *n* (%) *n* = 120	χ^2^/t	*p*	Cohens’d/Cramér’s V
Age	34.5 (4.20)	35.6 (5.41)	t = 1.65	0.101	Cohens’d = −0.22
Education Level					
Elementary School	7 (5.80)	4 (3.30)	χ^2^ = 4.38	0.112	Cramér’s V = 0.21
High School	76 (63.30)	64 (53.30)			
College/University	37 (30,80)	52 (43.30)			
Employment status					
Permanent Employment	67 (55.80)	99 (82.50)	χ^2^ = 20.21	0.001	Cramér’s V = 0.35
Temporary Employment	36 (30)	13 (10.80)			
Unemployed	17 (14.20)	8 (6.70)			
Number of family members					
3	86 (71.70)	30 (25)	χ^2^ = 61.27	0.001	Cramér’s V = 0.45
4	34 (28.30)	66 (55)			
5	0 (0)	24 (20)			

ASD: Autism Spectrum Disorder; TD: Typically Developing; *p*: *p* values; t: *t*-test; χ^2^: Chi-squared; *n*: number of participants.

**Table 3:** Differences in alexithymia levels between parents of children with ASD and parents of TD children.

	ASD M (SD) *n* = 120	TD M (SD) *n* = 120	t	*p*	Cohens’d
Difficulty describing emotions	16.61 (3.63)	12.56 (2.58)	9.97	0.001	1.29
Difficulty identifying emotions	23.7 (6.67)	14.8 (3.73)	12.75	0.001	1.65
External-oriented thinking	27.43 (2.89)	24.31 (2.77)	8.52	0.001	1.10
Alexithymia—total	67.74 (10.11)	51.68 (7.22)	14.16	0.001	1.83

ASD: Autism Spectrum Disorder; TD: Typically Developing; M: arithmetic mean; SD: standard deviation; *p*: *p* values; t: *t*-test; *n*: number of participants.

**Table 4:** Differences in the levels of family cohesion and flexibility, and family communication quality and satisfaction, between parents of children with ASD and parents of TD children.

	ASD M (SD) *n* = 120	TD M (SD) *n* = 120	t	*p*	Cohens’d
Balanced family cohesion	19.52 (2.61)	28.64 (2.58)	27.19	0.001	−3.51
Balanced family flexibility	19.84 (2.10)	27.56 (2.23)	27.61	0.001	−3.56
Unbalanced family cohesion scales					
Disengaged	20.89 (2.22)	11.06 (1.82)	37.46	0.001	4.84
Enmeshed	21.76 (2.13)	16.28 (2.45)	18.52	0.001	2.39
Unbalanced family flexibility scales					
Rigid	21.02 (2.25)	13.97 (1.81)	26.73	0.001	3.45
Chaotic	22.42 (3.38)	16.28 (2.10)	16.91	0.001	2.18
Family communication	29.45 (6.33)	42.40 (1.86)	21.5	0.001	−2.78
Family satisfaction	30.56 (7.80)	43.21 (2.24)	17.08	0.001	−2.20

Note. ASD: Autism Spectrum Disorder; TD: Typically Developing; M: arithmetic mean; SD: standard deviation; *p*: *p* values; t: *t*-test; *n*: number of participants.

**Table 5:** Differences in family resilience assessments between parents of children with ASD and parents of TD children.

	ASD M (SD) *n* = 120	TD M (SD) *n* = 120	t	*p*	Cohens’d
Family communication and problem solving	81.30 (12.46)	106.85 (11.39)	16.58	0.001	−2.14
Family cohesion	21.28 (3.17)	26.67 (2.49)	14.65	0.001	−1.89
Ability to find meaning in adversity	22.19 (3.07)	27.95 (2.53)	15.88	0.001	−2.05
Family resilience—total	41.59 (6.62)	53.72 (5.54)	15.21	0.001	−1.99

ASD: Autism Spectrum Disorder; TD: Typically Developing; M: arithmetic mean; SD: standard deviation; *p*: *p* values; t: *t*-test; *n*: number of participants.

**Table 6:** Differences in sources and intensity of parental stress between parents of children with ASD and parents of TD children.

	ASD M (SD) *n* = 120	TD M (SD) *n* = 120	t	*p*	Cohens’d
Child difficulty	11.69 (1.69)	5.23 (2.79)	21.69	0.001	2.80
Child’s lack of adaptation	10.71 (2.53)	2.38 (1.77)	29.47	0.001	3.82
Unfulfilled expectations	9.99 (2.03)	2.55 (2.47)	25.49	0.001	3.29
Parent attachment to child	9.71 (2.12)	4.37 (1.71)	21.45	0.001	2.77
Child discipline	9.97 (1.98)	3.88 (2.07)	23.24	0.001	3.01
Communication with child	10.08 (2.22)	3.88 (2.63)	19.71	0.001	2.55
Sense of incompetence	9.87 (1.92)	5.63 (2.19)	15.93	0.001	2.06
Lack of support	9.89 (1.97)	4.77 (2.63)	17.01	0.001	2.20
Parental role limitations	11.47 (1.72)	5.64 (2.53)	20.91	0.001	2.70
Spousal relationship	11.0 (2.21)	6.22 (2.29)	16.39	0.001	2.12
Financial situation	9.88 (2.36)	8.08 (1.90)	6.50	0.001	0.84
Demands from other roles	10.71 (1.93)	7.85 (2.32)	10.36	0.001	1.34
Child’s health	10.35 (2.10)	1.28 (1.29)	40.28	0.001	5.20
Parental stress—total	135.31 (17.87)	61.77 (17.58)	32.14	0.001	4.15

ASD: Autism Spectrum Disorder; TD: Typically Developing; M: arithmetic mean; SD: standard deviation; *p*: *p* values; t: *t*-test; *n*: number of participants.

**Table 7:** Differences in the cognitive emotion regulation strategies employed by parents of children with ASD and parents of typically developing (TD) children.

	ASD M (SD) *n* = 120	TD M (SD) *n* = 120	t	*p*	Cohens’d
Self-blame	14.73 (1.59)	8.96 (2.73)	19.96	0.001	2.58
Acceptance	14.08 (1.86)	14.92 (1.36)	3.99	0.001	−0.52
Rumination	11.17 (2.45)	11.62 (2.50)	1.41	0.160	−0.18
Positive refocusing	10.75 (1.71)	15.87 (1.30)	26.11	0.001	−3.37
Refocus on planning	12.45 (1.26)	18.14 (1.25)	35.16	0.001	−4.53
Positive reappraisal	12.31 (1.52)	17.28 (1.31)	27.1	0.001	−3.50
Putting into perspective	12.18 (1.98)	15.36 (1.34)	14.53	0.001	−1.88
Catastrophizing	14.64 (1.09)	8.54 (2.31)	26.18	0.001	3.38
Blaming others	12.68 (2.27)	11.58 (1.93)	4.04	0.001	0.52

ASD: Autism Spectrum Disorder; TD: Typically Developing; M: arithmetic mean; SD: standard deviation; *p*: *p* values; t: *t*-test; *n*: number of participants.

**Table 8:** Differences in the level of perceived social support between parents of children with ASD and parents of TD children.

	ASD M (SD) *n* = 120	TD M (SD) *n* = 120	t	*p*	Cohens’d
Support from family	3.92 (0.81)	5.95 (0.78)	19.77	0.001	−2.03
Support from friends	3.99 (0.65)	5.96 (0.55)	25.48	0.001	−1.97
Support from significant other	3.42 (0.91)	5.72 (0.94)	19.26	0.001	−2.30
Social support—total	3.78 (0.79)	5.88 (0.76)	23.75	0.001	−2.10

Note. ASD: Autism Spectrum Disorder; TD: Typically Developing; M: arithmetic mean; SD: standard deviation; *p*: *p* values; t: *t*-test; *n*: number of participants.

**Table 9:** Results from the scale measuring family’s experience with autism.

*n* = 120	Min.	Max.	M (SD)
Experience of being a parent of a child with ASD	33.00	50.00	44.16 (4.50)
Family life	29.00	40.00	34 (2.62)
Child’s development, understanding and social relationships	36.00	60.00	50.26 (6.87)
Child’s symptoms (feelings and behaviours)	30.00	37.00	34.09 (2.18)
Family experience with autism—total	136.00	182.00	162.52 (10.42)

Min.: minimal; Max.: maximum; M: arithmetic mean; SD: standard deviation; *n*: number of participants.

**Table 10:** The correlation coefficients between the family cohesion scale and predictors included in the regression analysis in the group of parents of children with ASD (*n* = 120).

	1	2	3	4	5	6	7	8	9
1. Family cohesion	1	−0.22 *	−0.19 *	0.20 *	0.26 **	−0.55 **	−0.19 *	−0.21 **	0.17 **
2. Sex		1	0.39 **	−0.66 **	−0.09	−0.25 **	0.13	−0.23 **	−0.09
3. Age			1	−0.30 **	0.25 **	0.04	0.08	−0.13	−0.19
4. Employment status				1	0.15	0.10	−0.18 *	0.11	0.12
5. Number of family members					1	0.30 **	0.08	0.15	0.40 **
6. Alexithymia						1	0.06	−0.08	−0.28 **
7. Social support							1	0.17	0.31 **
8. Self-blame								1	0.09
9. Ability to find sense in adversity									1

* *p* < 0.05; ** *p* < 0.01.

**Table 11:** The correlation coefficients between the family cohesion scale and predictors included in the regression analysis in the group of parents of TD children (*n* = 120).

	1	2	3	4	5	6	7
1. Family cohesion	1	−0.49 **	0.45 **	−0.37 *	−0.46 **	−0.18 *	0.20 *
2. Sex		1	−0.44 **	−0.58 **	−0.70 **	−0.59 **	0.36 **
3. Employment status			1	−0.21 *	−0.24 **	0.21 *	0.14
4. Self-blame				1	0.75 **	0.84 **	−0.39 **
5. Rumination					1	0.69 **	−0.27 **
6. Catastrophizing						1	−0.61 **
7. Refocus on planning							1

* *p* < 0.05; ** *p* < 0.01.

**Table 12:** The results of hierarchical regression analysis for the criterion of family cohesion with the following predictors: sex, age, parents’ employment status, number of family members, alexithymia, social support, parental stress, cognitive emotion regulation and family resilience. The results are shown separately for the families of TD children and families of children with ASD.

	Family Cohesion					
	ASD (*n* = 120)			TD (*n* = 120)		
	R^2^	ΔR^2^	β	R^2^	ΔR^2^	β
Step 1	0.13			0.29		
Socio-demographic characteristics						
Sex			−0.14			−0.36 **
Age			−0.22 *			n/p
Employment status			−0.01			0.28 **
Number of family members			0.32 **			n/p
Step 2	0.36	0.22		0.29	0.01	
Socio-demographic characteristics						
Sex			0.08			−0.37 **
Age			−0.26 **			n/p
Employment status			0.09			0.27 *
Number of family members			0.15			n/p
Alexithymia—TAS			−0.52 **			0.03
Step 3	0.48	0.13		0.44	0.16	
Socio-demographic characteristics						
Sex			−0.21 *			−0.25 *
Age			−0.23 **			n/p
Employment status			0.13			0.18 *
Number of family members			0.05			n/p
Alexithymia—TAS			−0.50 **			0.05
Social support			0.24 **			n/p
Cognitive emotion regulation						
Self-blame			−0.32 **			−0.47 **
Rumination			n/p			−0.39 **
Catastrophizing			n/p			−0.85 **
Refocus on planning			n/p			0.29 **
Family resilience						
Ability to find sense in adversity			−0.08			n/p

ASD: Autism Spectrum Disorder; TD: Typically Developing; *n*: number of participations; β: standardized regression coefficient; R^2^: adapted coefficient of multiple determination; ΔR^2^: change in coefficient of multiple determination; n/p: not a predictor; * *p* < 0.05; ** *p* < 0.01. TAS: Toronto Alexithymia Scale.

**Table 13:** The correlation coefficients between the family flexibility scale and predictors included in the regression analysis in the group of parents of children with ASD (*n* = 120).

	1	2	3	4	5	6	7
1. Family flexibility	1	0.46 **	0.22 *	−0.31 **	0.49 **	−0.30 **	−0.42 **
2. Sex		1	0.39 **	0.16	−0.09	0.13	0.39 **
3. Age			1	0.09	0.08	−0.13	0.10
4. Parental stress				1	−0.18 *	−0.01	−0.07
5. Social support					1	0.17	0.09
6. Self-blame						1	0.52 **
7. Catastrophizing							1

* *p* < 0.05; ** *p* < 0.01.

**Table 14:** The correlation coefficients between the family flexibility scale and predictors included in the regression analysis in the group of parents of TD children (*n* = 120).

	1	2	3	4	5	6	7	8	9	10	11
1. Family flexibility	1	−0.23 *	−0.22 **	0.22 *	0.26 **	−0.28 **	0.32 **	−0.18 *	−0.19*	0.18 *	0.29 **
2. Sex		1	0.29 **	0.03	0.05	−0.70 **	−0.25 **	−0.59 **	−0.37 **	−0.01	0.08
3. Age			1	−0.50 **	0.31 **	−0.19 *	−0.03	−0.35 **	−0.03	0.01	0.06
4. Education level				1	0.03	0.03	−0.01	0.01	0.12	0.14	0.03
5. Social support					1	0.11	0.03	−0.01	−0.12	0.16	0.29 **
6. Rumination						1	0.05	0.48 **	0.13	0.02	−0.04
7. Putting into perspective							1	−0.26 **	−0.32 **	0.05	0.07
8. Catastrophizing								1	−0.11	−0.02	−0.02
9. Blaming others									1	0.06	−0.02
10. Family communication and problem solving										1	0.75 **
11. Family cohesion											1

* *p* < 0.05; ** *p* < 0.01.

**Table 15:** The results of hierarchical regression analysis for the criterion of family flexibility with the following predictors: sex, age, education level, parental stress, social support, cognitive emotional regulation and family resilience. The results are shown separately for the families of TD children and families of children with ASD.

	Family Flexibility					
	ASD (*n* = 120)			TD (*n* = 120)		
	R^2^	ΔR^2^	β	R^2^	ΔR^2^	β
Step 1	0.20			0.08		
Socio-demographic characteristics						
Sex			0.45 **			−0.22 *
Age			0.04			−0.05
Education level			n/p			0.20 *
Step 2	0.53	0.34		0.60	0.53	
Socio-demographic characteristics						
Sex			0.46 **			−0.04
Age			0.04			0.04
Education level			n/p			0.27 **
Parental stress			−0.17 *			−0.46 **
Social support			0.33 **			−0.09
Cognitive emotion regulation						
Self-blame			−0.35 **			n/p
Rumination			n/p			0.08
Catastrophizing			0.01			−0.40 **
Blaming others			n/p			−0.37 **
Putting into perspective			n/p			0.65 **
Family resilience						
Family communication and problem solving			n/p			0.17
Family cohesion			n/p			0.50 **

ASD: Autism Spectrum Disorder; TD: Typically Developing; *n*: number of participations; β: standardized regression coefficient; R^2^: adapted coefficient of multiple determination; ΔR^2^: change in coefficient of multiple determination; n/p: not a predictor; * *p* < 0.05; ** *p* < 0.01.

## Data Availability

The datasets used and/or analysed during the current study are available from the corresponding author on reasonable request.
